# Histologic and molecular analysis of patient derived xenografts of high-grade serous ovarian carcinoma

**DOI:** 10.1186/s13045-016-0318-6

**Published:** 2016-09-21

**Authors:** Ruifen Dong, Wenan Qiang, Haiyang Guo, Xiaofei Xu, J. Julie Kim, Andrew Mazar, Beihua Kong, Jian-Jun Wei

**Affiliations:** 1 Department of Pathology, Northwestern University School of Medicine, Feinberg 7-334, 251 East Huron Street, Chicago, IL 60611 USA; 2 Department of Obstetrics and Gynecology, Qilu Hospital, Shandong University, 107 Wenhuaxi Road, Jinan, Shandong 250012 China; 3 Department of Obstetrics and Gynecology, Northwestern University Feinberg School of Medicine, Chicago, IL USA; 4 Department of Pharmacology, Feinberg School of Medicine and Chemistry of Life Processes Institute, Northwestern University, Chicago, IL USA; 5 Institute of Genetics, Shandong University School of Medicine, Jinan, Shandong China

**Keywords:** High-grade serous carcinoma, Patient-derived xenograft, Intrabursal engraft histology, Immunohistochemistry

## Abstract

**Background:**

Patient derived xenografts (PDX) are generated by transplanting the original patient’s tumor tissue into immune-deficient mice. Unlike xenograft models derived from cell lines, PDX models can better preserve the histopathology from the original patient and molecular pathways. High-grade serous carcinoma (HGSC) is a deadly form of ovarian/fallopian tube cancer whose response to current chemotherapies varies widely due to patient variability. Therefore, a PDX model can provide a valuable tool to study and test treatment options for each individual patient.

**Methods:**

In this study, over 200 PDX tumors from nine HGSC were analyzed to investigate the nature and behavior of PDX tumors originating from HGSC. PDX tumors were serially passaged (from P0 to P4) and tumors were grafted orthotopically under the ovarian bursa or subcutaneously.

**Results:**

Comparative analysis of the histology and molecular markers of tumors from over 200 PDX tumor-bearing mice, revealed that the tumors maintained similar histologies, stem cell populations, and expression for the majority of the tested oncogenic markers, compared to the primary tumors. However, a significant loss of steroid hormone receptors and altered expression of immunoresponsive genes in PDX tumors were also noted.

**Conclusion:**

Our findings provide substantial new information about PDX tumor characteristics from HGSC which will be valuable towards the development of personalized therapy and new drug development for HGSC.

**Electronic supplementary material:**

The online version of this article (doi:10.1186/s13045-016-0318-6) contains supplementary material, which is available to authorized users.

## Background

Epithelial ovarian cancer (EOC) has a disproportionately high mortality rate in comparison to other female malignancies [1]. According to the American Cancer Society, 21,290 women will be newly diagnosed with EOC and 14,180 women will succumb to this disease in 2015 [2]. Of all EOC, high-grade serous ovarian carcinoma (HGSC) is the most lethal ovarian cancer histotype [3], which accounts for nearly 75 % of all EOC-related mortality. Most HGSC respond to combined paclitaxel and carboplatin (Tax/Carp) chemotherapy after surgical treatment. However, almost all HGSC relapse and eventually become chemoresistant. Long-term treatments for HGSC remain a challenge, and the overall survival rate has not been significantly improved in the past several decades.

Traditionally, the assessment of experimental cancer therapies using the established ovarian cancer cell lines have many limitations and cannot truly reflect the complexity and interpatient variation of ovarian cancer. Patient-derived xenograft (PDX) or a xenopatient is a system in which a portion of a patient’s tumor, obtained either by surgical resection or biopsy, is transplanted in immunodeficient mice and allowed to propagate without any in vitro manipulation. Tumors can be engrafted heterotopically or orthotopically [4], and both have been found to mimic the human tumors [5, 6], thus allowing for better prediction of a patient’s response to chemotherapy [7]. A recent study reporting the largest PDX collection to date, revealed that subcutaneous (SC) PDX were reliable in predicting for clinical activity [6]. Although the impact and degree of genetic alterations that occur with each tumor passage remains unclear [4], PDX models mostly retain the principal histologic and major genetic characteristics of their donor tumor and have been used for preclinical drug evaluation, biomarker identification, biologic studies, and personalized medicine strategies [8].

EOC tumors are highly heterogeneous, with variable responses to standard chemotherapies emphasizing the need for PDX models to study EOC diversity and aid in novel therapeutical development [9]. It is also important to establish the preservation of PDX tumor characteristics from the primary tumor. In this study, we examined and compared primary HGSC with serial passages of PDX tumors with regard to histology, stem cells, and expression of molecular markers.

## Methods

### Tissue samples

Fresh primary ovarian carcinoma tissues were obtained from chemotherapy naïve ovarian cancer after resection at the Prentice Women’s Hospital of Northwestern University from September 2013 to June 2014. Prior to surgery, written informed consent for tissue acquisition was obtained and nine consecutive cases of HGSC were collected. All tumors were collected and engrafted within 2 h post resection. Normal fallopian tube tissues were collected as normal control. Each case was reviewed by pathologists to confirm the diagnosis. The collection of human tissue specimens and the PDX mouse protocol were approved by the Institutional Review Board and Institutional Animal Care and Use Committee at Northwestern University. The clinical and pathological features of patients are summarized in Table 1.

**Table 1:** Main clinical and pathological characteristics of tumor tissues

Case ID	Subtype	Stage	Surgical procedure	Tumor size (cm)
OVCA4	HGSC	T3C	TAHBSO	7
OVCA5	HGSC	T3C	TAHBSO	5
OVCA6	HGSC	T3C	TAHBSO	14
OVCA7	HGSC	T3B	TAHBSO	13.1
OVCA8	HGSC	T3C	TAHBSO	5
OVCA9	HGSC	T3C	TAHBSO	12
OVCA10	HGSC	T3A	TAHBSO	9
OVCA12	HGSC	T3C	BSO	1.4
OVCA13	HGSC	T3C	TAHBSO	6

*HGSC* high grade serous carcinoma, *TAH* total abdominal hysterectomy, *BSO* bilateral salpingo-oophorectomy

### Microarray analysis

Total RNA was isolated using the Trizol reagent (Invitrogen) and PureLink RNA Mini Kit (Ambion) according to manufacturer’s instructions. RNA quantity was assessed by NanoDrop 1000 spectrophotometer, Agilent 2100 bioanalyzer, and PCR bioanalysis and samples with an RNA integrity number (RIN) that scored higher than 8.0 were used. Expression profiling was performed using a HumanHT-12 v4 Expression Beadchip (Illumina) at the Northwestern Genomic Core Facility. Expression data were normalized using the median normalization. After normalization, significant differentially expressed mRNAs were identified through volcano plot filtering. Finally, hierarchical clustering was performed to show distinguishable mRNA expression profiling among samples.

### DNA extraction and P53 mutation analysis

The genomic DNA of nine primary cases was extracted and purified using the QIAamp DNA FFPE Tissue Kit (QIAGEN) according to the manual. P53 exon4-9 mutation analysis was conducted as previously described [10]. In brief, 50 ng genomic DNA was amplified by PCR with HotStarTaq Master Mix (QIAGEN). PCR products were purified using the Gel Extraction and PCR Clean-Up Kit (Clontech). DNA sequencing of the purified DNA products was performed in the NU core facility by the ABI 3730 High-Throughput DNA Sequencer (Applied Biosystems) at the Genomic Core Facility. The mutations and variations were analyzed using DNASTAR Lasergene 9 software. Detailed information of primers used for the amplification and sequencing are listed in Additional file 1: Table S1.

### RNA isolation and quantitative real-time PCR

RNA isolation and quantitative real-time PCR (qPCR) was conducted similarly as before [11]. Briefly, total RNA was extracted from fresh tissues with Trizol reagent (Invitrogen). The reverse transcription reaction was performed using Mir-X™ miRNA First-Strand Synthesis Kit (Clontech). QPCR was performed with Fast SYBR® Green Master Mix (Invitrogen) with StepOne Plus Real-Time PCR System (Applied Biosystems).

### Xenograft of tumor tissues

Eight-to-twelve-week-old female adult non-obese diabetic (NOD)-scid IL2Rγ^*null*^ or NSG mice (The Jackson Laboratory) were used. Mice were maintained in laminar flow rooms, maintaining consistent temperature and humidity and were given free access to water and a normal diet. Mice were housed for 14 h light and 10 h dark cycle. Experiments were approved by the Institutional Animal Care and Use Committee of Northwestern University. Xenografted tissues were labeled as passage 0 (P0), P1, P2, etc. depending on the number of passages from the initial tumor.

#### Subcutaneous (SC) xenograft

For the first generation (P1) of xenografted, fresh tumor tissues (P0) collected from patients were cut into small (~3 × 3 × 2 mm) fragments, and then two tissue fragments were subcutaneously xenografted to each dorsal? flank of a NSG mouse.

For other generations (≥P2), tumor tissues were cut into small pieces (~2 × 2 × 2 mm). Then, two to four tissue fragments were subcutaneously xenografted into two dorsal? flanks of NSG mice. First, mice were anesthetized by intraperitoneal injection of ketamine/xylazine (90/8 mg/kg), and the mice were shaved on the back where the surgery would occur, and the site was disinfected with providone iodine prep pads and alcohol swab (70 % isopropyl alcohol). An one cm in length incision was made in the skin at the midline of the mouse back, and four separate tumor fragments were put into the upper left, upper right, lower left, and lower right of back, accordingly. After implantation, the skin was sutured, and mice were revived.

#### Intrabursa (IB) xenograft

Tumor tissues were cut into small pieces (~1 × 1 × 1 mm) and grafted onto the left side of the ovarian intrabursa of adult female NSG mouse hosts. The procedure of implantation for IB xenograft is the same as previously described [12]. Mice were anesthetized by intraperitoneal injection of ketamine/xylazine (90/8 mg/kg) and the mice were shaved on the back where the surgery would occur, and the site was disinfected with providone iodine prep pads and alcohol swab. A 1 cm in length incision was made in the skin just laterally to the midline of the lower back, and the ovary was visible under the muscle layer. After pulling out the left ovary, the ovarian bursa would be identified. A tiny hole was made under the surgical microscope, and the tumor fragment was grafted into the intrabursa. The ovary was put back in place, and if no bleeding was noted, the incision on the muscle layer and body wall was closed separately. Mice were given analgesics (meloxicam) for pain management for 2 days post-surgery.

### Necropsy

Mice were sacrificed when the tumor size reached 1.5 cm in diameter or ascites emerged. Body weight was measured, and mice were sacrificed by intraperitoneal injection of ketamine/xylazine (90/8 mg/kg).

After dissection of the tumors, tumor size was documented by measuring tumor diameters. Then, tumor volume was calculated according to the formula TV (mm3) = *a* × *b* × *c* × л/6, where *a* is the length, *b* is the width, and *c* is the height. All organs in the peritoneal, pelvic, thoracic, and cranial cavities were dissected out and checked for possible metastasis. Numbers of metastasis was documented and images of metastasis were photographed. Female reproductive tissues including the bilateral ovaries and uterus were isolated and fixed in modified Davidson’s fixative. The other organs including brain, heart, lungs, liver, pancreas, spleen, kidneys, stomach, intestine, cecum, rectum, omentum, and diaphragm were also collected and fixed in modified Davidson’s fixative. All fixed tissues were processed, embedded in paraffin, and sectioned and then hematoxylin and eosin (H&E) staining was performed for histologic examination.

### Subcutaneous tumor growth

Four tumor fragments were subcutaneously xenografted into two mice. Tumor growth was monitored by measuring tumor diameter every 2 weeks. Tumor volume was calculated according to the formula TV (mm3) = *a* × *b*^2^ × π/6, where *a* is the longest diameter, and b is the shortest diameter. Mouse was euthanized when a tumor reached 1.5 cm in diameter.

### Tissue microarray and immunohistochemistry

Tissue cores were collected from tumors for tissue microarray and represented in duplicate. Tissue microarrays were sectioned at 4 μm in thickness. Tissue microarray slides were deparaffinized in xylene and rehydrated in a graded series of ethanol. After antigen retrieval, all immunohistochemical staining was performed on a Ventana Nexus automated system. In brief, endogenous peroxidase activity was blocked with 3 % hydrogen peroxide. After blocking in 1.5 % normal goat serum for 30 min at room temperature, slides were then incubated overnight at 4 °C with primary antibodies in a humid chamber. Staining was detected with I-View 3,3′-diaminobenzidine (DAB) detection system.

Semiquantative immunointensity was scored as 0 (negative), 1 (weak), 2 (moderate) and 3 (strong) and percentage was showed as %. Immunoreactivity for HMGA2, MTSS1 and P16 was scored for intensity only. Immunoreactivity for Ki67, P53, P21, ER, PR, ALDH1, CD24, and CD133 was scored for percentage only. Antibodies used for this study were listed in Additional file 1: Table S2.

### Statistical analysis

The software SPSS V20.0 was used for statistical analysis. All data were presented as means and standard errors. Student’s *t* test and one-way ANOVA analysis were used to determine significance. *P* < 0.05 was considered statistically significant.

## Results

### Strategy for establishment of patient derived xenografts (PDX) for HGSC

**Fig. 1:**
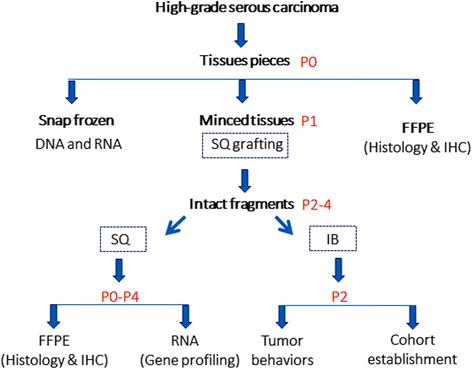
A Sketch diagram illustrating the work flow for PDX for human HGSC. HGSC tissue were collected from patients (defined as P0) and divided into three aliquots for PDX (small (~3 × 3 × 2 mm) fragments of tissues for subcutaneous (SQ) xenograft as P1), for snap frozen (for later DNA and RNA extraction) and for formalin-fixed and paraffin-embedded (FFPE) preparation (for TMA and immunohistochemistry (IHC) analysis). The extended passages (P2-P4) of tumor xenografts were established for gene profiling analysis and further histological and molecular analysis

**Fig. 2:**
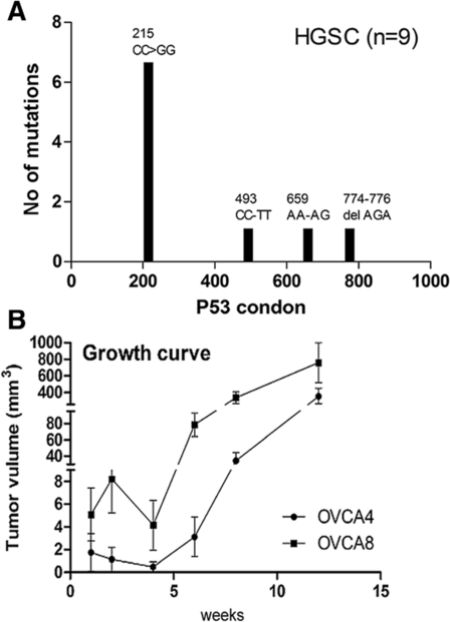
Molecular analysis of *TP53* mutations and tumor growth rate of PDX tumor. **a** Distribution (x-axis) and frequency (y-axis) of *TP53* mutations in nine HGSC detected. **b** The growth curve of two representative HGSC engrafted subcutaneously (SQ) from OVCA4-P2 and OVCA8-P2. The tumor volume was calculated by measuring the diameter of SQ tumors

**Fig. 3:**
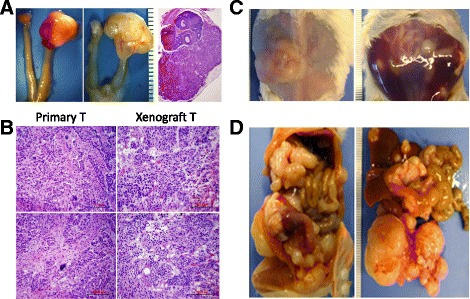
The patterns of tumor growth and metastasis in intrabursal engrafting of HGSC. **a**. Photomicrographs illustrate gross appearance of intrabursal engrafting of HGSC at the end of experiment (*left*) and hematoxylin/eosin stained section (*right*). **b** Photomicrographs of frozen sections for a side-by-side comparison of primary and xenograft tumors (H/E stain). **c**, **d** Photomacrographs illustrate examples of ascites (**c**) and metastasis (**d**) in mice with intrabursal engrafting of HGSC. **b** Photomicrographs show histologic and cytological similarity of primary and engrafted HGSC performed by onsite frozen section and hematoxylin and eosin stain

**Fig. 4:**
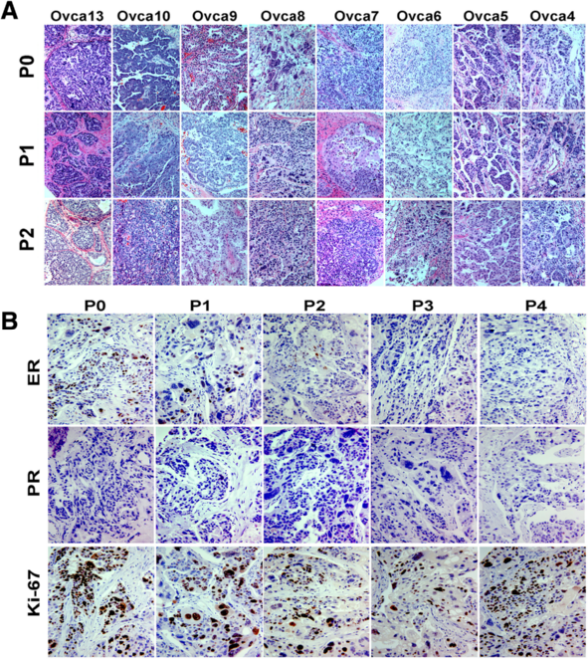
Histology and immunohistochemistry analysis of primary (P0) and PDX HGSC (P1-4). **a** Photomicrographs of tissue sections from primary (P0), passage 1 (P1) and passage 2 (P2) in each of nine high grade serous ovarian carcinoma (Ovca 4-13). **b** Photomicrographs illustrate an example of immunoreactivity for estrogen receptor (ER), progesterone receptor (PR) and Ki-67 (cell proliferation marker) in primary (P0) and engrafted carcinoma of passage 1 to 4 (P1-4)

In this study, we designed a standard for workflow to establish PDX for HGSC and to evaluate the PDX tumor biology (Fig. 1). A frozen section evaluation was performed to confirm the histologic type of HGSC and to define viable tumor tissue for xenografting. All evaluated primary tumor tissues were divided into three aliquots: (defined as P0) (i) PDX, (ii) freezing for molecular studies, and (iii) histology by formalin-fixation and paraffin-embedding (FFPE). The frozen tissues were used to extract DNA and RNA for P53 status analysis. All nine cases had p53 mutations and the frequency of mutations at specific codons is shown in Fig. 2a. For PDX, small (~3 × 3 × 2 mm) fragments of tumor tissues were implanted subcutaneously (SQ) in two mice as P1. After the tumors reached a size of 1.0 to 1.5 cm, they were removed and reevaluated by frozen section (Fig. 3b). Tumor fragments (2 × 2 × 2 mm) were then implanted SQ for a serial passage of tumors (P2-P4). As shown in Fig. 2b, PDX tumors became smaller at 2 to 4 weeks and then grew steadily from 6 to 12 weeks. Global gene profiling analysis was done in P0 and P2 tumors to compare the primary and xenograft tumors. All PDX tumors were prepared for FFPE and examined histologically and portions of P0 and P1-4 PDX tumors were collected to prepare a tissue microarray (TMA) for immunohistochemistry analysis (Additional file 1: Figure S1). The specific focus was on how the biomarkers of HGSC related to tumor signature, proliferation and invasion, and tumor stem cells (Fig. 4 and Additional file 1: Table S4).

### Establishment of intrabursa (IB) PDX

IB implantation of epithelial ovarian cancer has been performed successfully in our lab using ovarian cancer cell lines [12]. The bursa provides the ovarian microenvironment for the primary HGSC. The IB implantation of tumors resulted in the growth of the PDX tumors, ascites accumulation, and wide spread metastasis to the reproductive organs, pelvic wall, and other abdominal organs including the liver, pancreas, spleen, kidney, omentum, and diaphragm (Fig. 3c, d) which were similar to that of human HGSC. Histological examination showed that PDX tumors maintained similar morphology and growth pattern to the original tumors (Fig. 3b).

### Histologic analysis of different passages of PDX HGSC tumors

**Fig. 5:**
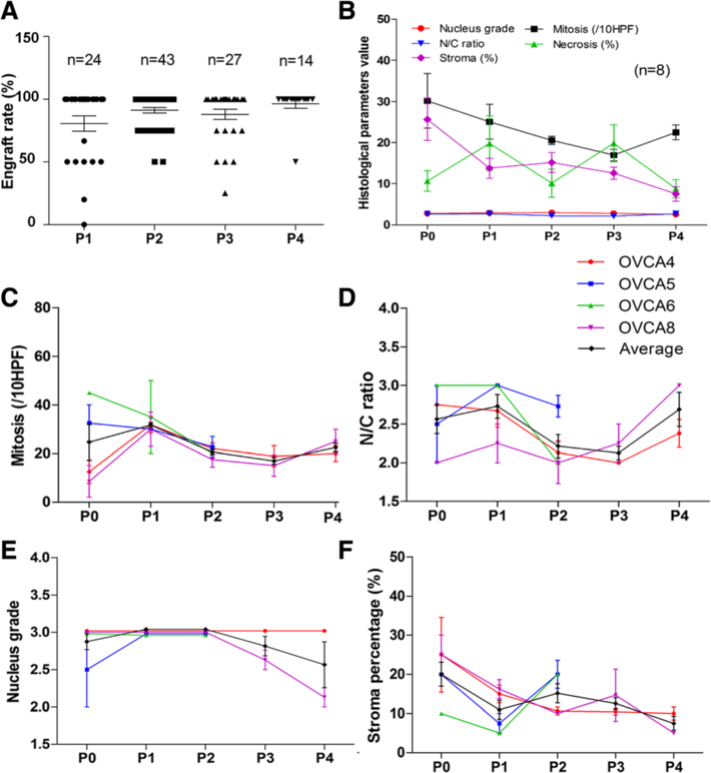
Histological analysis of primary (P0) and passage 1 to 4 (P1-P4) engrafted HGSC. **a** *Dot plots* illustrate the success rate of engrafted tumor tissues from P1-P4 (calculated based on the tumor numbers of engraft ones and survival ones). Each *dot* represents one engraft and number of engrafts listed above (n). **b** The general patterns of five selected histological parameters in P0 to P4 measured from 8 cases. (**c**-**f**) Histologic analysis of four selected parameters (**c** mitosis; **d** nuclear and cytoplasmic rate (N/C); **e** nuclear grade and **f** stromal %). Available data from four representative cases (OVCA4, 5, 6 and 8) were used for the analysis. At least three engrafts from each case were measured for mean (*solid lines*) and standard errors (*small t-bars*)

The success of engraftment (SOE) for primary ovarian cancer has not been defined. Based on a recent study of 241 cases, 12 months was the cutoff and an overall 74 % engraft rate was observed [13]. Our current study on HGSC showed a slightly higher SOE at P1 (Fig. 5a), most likely due to the pre-evaluation of tumors using frozen sections before grafting (Fig. 2b). Of the nine cases, only one case (OVCA12) did not generate palpable tumors by SC at P1. In our study, SOE reached 90 % at P2 and even higher in P3 and P4 (Fig. 5a).

All P0 to P4 HGSC were collected and subjected to histologic evaluation (Fig. 4a, Additional file 1: Table S3). Growth pattern, nuclear grade, nuclear cytoplasmic ratio (N/C ratio), tumor stroma, mitotic index, and tumor necrosis were analyzed for the eight cases that were successfully engrafted at all passages (Fig. 5b, Additional file 1: Table S3). Four cases with four passages (P4) were further analyzed (Fig. 5c–f). The mitotic index varied widely among primary HGSC tumors (Fig. 5a). By P4, the mitotic index stabilized to approximately 20–30 mitosis/10 HPF (Fig. 5c). N/C ratio varied from case to case, but each of them maintained a similar ratio throughout P0 to P4 (Fig. 5d). The stroma content reduced with passage in the PDX tumors (Fig. 5f). Primary tumors (P0) consisted of about 25 % of stroma and by P4, stroma made up 10 % of the tumor. Tumor necrosis was present in all passages. The nucleus pleomorphism and grade in P2 were slightly higher than in P0 but returned in P4 (Fig. 5e).

### Molecular analysis of HGSC in original and PDX tumors

**Fig. 6:**
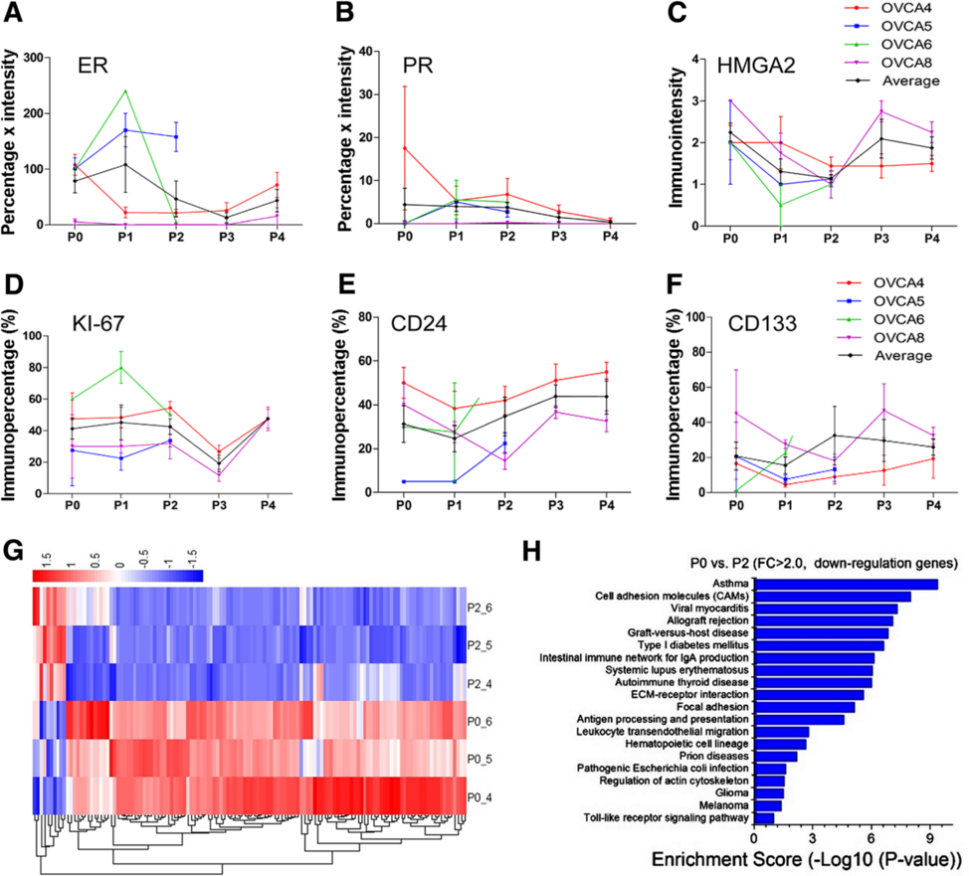
Molecular analysis of the selected immunomarkers and global gene profiling in P0 and P2. **a**–**f** Semiquantitative analysis of ER (**a**), PR (**b**), HMGA2 (**c**), KI-67 (**d**), CD24 (**e**), and CD133 (**f**) from four cases. At least three engrafts from each case were measured for mean (*solid lines*) and standard errors (*small t-bars*). **g** Heatmap shows over 130 significantly dysregulated (>twofold) genes between P0 and P2 HGSC. The color *red* represents overexpressed and *blue* indicates genes that are under expressed in P2. **h** The pathway analysis listed the altered functional pathways in P2 tumors in comparison to P0. The length of *blue bars* indicates the enrichment scores in each pathway

To evaluate the molecular differences of primary and engrafted tumors, we prepared a TMA to include all engrafted tumors (124 tumors) generated from eight cases (Additional file 1: Figure S1) and examined the biomarkers which were relevant to HGSC, including tumor signature markers (ER, PR, P53, P16), tumor proliferation (Ki-67, P21, P16), invasion (HMGA2), and stem cells (ALDH1, CD24, CD133) (Fig. 6a-f and Additional file 1: Table S4). Cell proliferation was measured by Ki-67 index (Fig. 6d). As shown in Figs. 4b and 6a, b, there was a steady decrease of ER and PR expression from P1 to P4. The immunopercentage of ER was close to 80 % at P0, it dropped to below 50 % at P4. Similarly, the immunopercentage of PR ranged 5–10 % in P1 and P2 but disappeared in P4. Immunoreactivity for HMGA2 was high in P0, slightly reduced in P1 and P2, and restored in P3 and P4 engrafts (Fig. 6c).

It has been shown that tumor stem cells contribute to the tumors’ growth and resistance to therapy in HGSC [14]. In this study, we examined three stem cell biomarkers (ALDH1, CD24 and CD133) in P0 to P4 HGSC (Fig. 6e and f). ALDH1 had very low immunoreactivity in most P0 to P4 tumors, and it was not informative for further evaluation (data not shown). Based on semiquantitative analysis of CD24 and CD133, we observed a high level of immunoreactivity for CD24/CD133 in all cases. No significant change of CD24/CD133 cell population in primary and engrafted tumors was observed. This suggests that HGSC may have a stable number of stem cells in both primary and xenograft tumors.

To further evaluate the difference of gene expression between primary and engrafted HGSC, we conducted the global gene profile analysis in P0 and P2. The difference of the gene expression between P0 and P2 may represent the true barrier/bottleneck for the development of future therapeutic strategy. A total of 130 genes were differentially expressed between P0 and P2 tumors (>twofold, ANOVA *p* < 0.05) (Fig. 6g). Pathway analysis revealed that three major pathways were altered in the engrafted tumors: (1) immune modulated pathways for autoimmune system, graft-versus-host disease (GVHD), allograft rejection; (2) extra-cellular matrix interaction; and (3) cell adhesion molecules (CAMs) (Fig. 6h and Additional file 1: Table S5). This finding implies that dysregulated genes are mostly related to the engrafted microenvironment. No significant change in genes relating to oncogenic properties of HGSC was found.

## Discussion

Attempts for PDX in ovarian cancer have existed for decades. In 1977, Davy et al. was the first to conduct subcutaneous (SQ) heterotransplants of ovarian cancer tissue into nude mice [15]. In 1984, Stratton et al. applied a subrenal (SR) xenograft model for ovarian cancer cells from ascites cells with better success rates [16]. Several years later, Ward et al. established xenografts in nude mice by intraperitoneal (IP) injections of fresh primary tumor slurries or of small tumor refractions derived from patient specimens [17]. Several attempts for testing orthotopic ovarian cancer models were also reported, including intrabursal (IB) [18] and an intra-gonadal fat pad of mice [19]. Since then, many studies using ovarian cancer PDX models were reported [20–24], including serous carcinoma [22, 23, 25–28], clear cell ovarian cancer models [29, 30], mucinous and an endometrioid ovarian cancer model [19]. Lee et al. established ovarian cancer PDX models that included almost all epithelial ovarian cancers [31]. The largest known living tumor bank of PDX for ovarian cancer involved 241 cases of patients, including ovarian, peritoneal, and fallopian tube cancer [13]. The model resulted in a 74 % engraft rate in SCID mice. The success rate in establishing PDX varied, depending on tumor type, tissue quality, site of transplantation, and strain of mouse [32]. Overall, the engrafting rate in NOD/SCID or NSG models is higher than other strains [8]. It seems that those successfully engrafted tumors may have an aggressive clinical course [13, 33]. In this study, we observed an average of 70 % engrafted rate of HGSC in P1 and 90–100 % engrafted rate in P2-4. Apparently, with the proper techniques and freshly collected tumor tissue, PDX for HGSC can be readily used as a reliable model for PDX. However, for many cases, they take months to grow visible tumors, and this is a major obstacle for the urgent needs for clinical trials and therapeutical purposes.

IP and orthotopic models can mimic the patients of the metastasis pattern or ascites formation [19, 34]. The tumors could metastasize to the ovaries, bowel, omentum, liver, mesentery, spleen, pancreas, and diaphragm [13, 35]. In this study, we engrafted P1 tumors SQ and let them grow to sizable tumor masses and then we engrafted P2 tumors intrabursally as described in the methods and result. In such intrabursa engrafts, we observed “primary” and “metastatic” tumors which are similar to the growth patterns seen in human ovarian cancer (Fig. 3). This valuable model can be potentially used for evaluating tumor growth behavior in early and later stage disease by responding to the therapeutic modality.

PDX of ovarian cancer can be passaged and retransplanted for up to six generations [15, 20, 36], and some of the tumors by IP injection were passaged to 24 generations [37]. All these studies indicate a reliable model of PDX for ovarian cancer. In this study, we used the NSG strain, and we found the engrafting rate in P1 was about 70 % and in P2 was 90 %. Failure rate in P3 and P4 was even lower (Fig. 5a). P2 could be the best tumor model for potential therapeutical purposes as it has a high rate of engraft success, shorter engraft time, and comparable histology and tumor related markers to primary tumors (Figs. 5 and 6).

One essential determinate of the validity of the PDX tumors is the maintenance of similar histologic and molecular characteristics of the repeated passages of PDX tumors to primary tumors. Several studies suggested that ovarian cancer PDX can maintain similar architectures and growth pattern as primary tumors [20, 25, 31, 37, 38], but specific details were lacking. Our current study provides a comprehensive analysis for each of the specific histologic features, such as nuclear grade, nuclear cytoplasmic ratio, mitotic index, tumor necrosis, and tumor/stromal ratio between the primary and passaged tumors of HGSC. Our quantitative analysis and assessment of these histologic features can be a valuable baseline for our understanding of the nature of the HGSC PDX tumors. Of note, our data further supports that HGSC PDX tumors (P1-P4) maintain similar but more uniform histologic features than primary tumors (Figs. 4, 5, and 6).

The published data suggested that both primary and PDX tumors maintained a similar molecular expression pattern [35, 38]. To test whether these findings apply to HGSC, we compared the gene expression between P0 and P2. Among 11 selected markers that are relevant to HGSC, a significant down-regulation of ER and PR expression was noted in P2 PDX tumors in comparison to P0 (Figs. 4 and 6). This change may have an impact on some anti-steroid hormonal therapies. Global gene profiling analysis revealed that the genes involving autoimmune, cell adhesion, and the extracellular matrix were significantly dysregulated in P2 PDX tumors (Fig. 6). These findings suggest that the graft microenvironment can influence the immune modulation, cell-cell interaction, and stromal reaction. The latter may result in different responses to immune therapies between PDX and primary tumors. No significant change of oncogenic pathways commonly dysregulated in HGSC was seen and the findings may be ideal for targeted therapies for oncogenic or tumor suppressor pathways.

Proliferation and mitotic index seem to be higher in PDX tumors than primary ones [31]. We found that the cell proliferation and mitotic index varied widely among different cases, but there was a tendency to be synchronized to a relatively stable proliferation index in P2-P4 tumors (Fig. 5). Ovarian cancer stem cells (CSC) in PDX tumors were examined in several studies [21, 23, 39]. Due to different techniques and markers selected, the interpretation of CSC remains controversial. Based on semiquantitative analysis of CD24 and CD133, we found that both primary and PDX tumors in HGSC maintained relatively similar numbers or ratios of CSC populations (Fig. 5, Additional file 1: Table S4).

It seems that current chemotherapies used in the clinic may be as effective in treating PDX tumors as primary ovarian cancer [13, 22]. For example, PDX tumors respond to cisplatin or carboplatin similar to primary tumors [13, 25, 35, 40]. Primary human platinum-resistant HGSC were also established as PDX, and novel agents such as notch signaling pathway inhibitor [26], PARP inhibitor olaparib (AZD2281) [41], and the DNA minor groove binder lurbinectedin [25] have been tested [29, 42]. To achieve the goal for the clinical usage of PDX, thorough evaluation of PDX tumors, including tumor growth behavior, histology, molecular alterations, and stem cell dynamics will provide basic parameters for its potential application to existing or new therapeutic targets.

There are several published data on ovarian cancer PDX models, but the results vary widely among studies due to different histologic subtypes, implantation site, and passage and analysis platforms. To use PDX as a tool for potential therapeutical purposes, it is very important to compare the histologic and molecular difference, stem cell change, and growth behavior in different microenvironments between primary and engrafting tumors. Therefore, an in-depth analysis of PDX tumors should include the engrafting site, passage time and level, microenvironment, and primary and metastatic tumors. To this end, we compared five of the most recent and similar studies and the results are summarized in Table 2 [13, 20, 22, 35]. We listed the major parameters and findings which are necessary for the evaluation of PDX tumors. This check list may aid in future studies and for potential clinical applications. Through thorough evaluation of histologic and molecular differences between primary and xenograft tumors, PDX models may provide a novel approach and angle for the evaluation of HGSC tumors’ behavior and biologic features. The findings may further benefit towards designing optimal passages of PDX tumors to meet the needs for personalized medical treatments.

**Table 2:** Biomedical and pathology comparison of most recent studies in ovarian cancer PDX models

		Ricci et al. (2014) [35]	Weroha et al. (2014) [13]	Dobbin et al. (2014) [20]	Topp et al. (2014) [22]	Current study
No. cases		34	168	34	12	9
Tumor types		All EOC types	All EOC types	All EOC types	High-grade serous	High-grade serous
Implantation site	SQ	Yes	No	Yes	Yes	Yes
	IP	Yes	Yes	Yes	No	No
	IB	Yes	No	No	Yes	Yes
Take rate (%)		25	74	85.3 (SC), 22.2 (IP)	83	>90
Passage time (weeks)	Average	Not mentioned	Not mentioned	10 weeks	Not mentioned	6–12 weeks
Passage attempts		P1->6	P1	P1-6	P1	P1-4
Stem cell analysis		No	No	ALDH1, CD44,CD133	No	ALDH1, CD44,CD133
Histology comparison		Yes	Yes	No	No	Yes
Immunohistochemistry analysis	ER/PR	No	No	No	Yes	Yes
	KI67	No	Yes	Yes	Yes	Yes
Mutation analysis	P53	Yes	No	No	Yes	Yes
Gene profile	P0	Yes	Yes	No	No	Yes
	P1-x	Yes	P1	No	No	P2

*EOC* epithelial ovarian cancer

## Conclusions

In summary, we established the heterotopic and orthotopic PDX for HGSC in this study. The histological and molecular analysis provided valuable information for the future use of HGSC PDX. Our findings support the complexity of ovarian tumor histology, stem cells, and molecular characteristics, indicating a need for a PDX model in order to develop personalized medical treatments for this deadly disease.

## Additional file

Additional file 1: Table S1. Primers and sequences used in this study. **Table S2.** Information of antibodies used in IHC assay. **Table S3.** Histology pattern between different passages. **Table S4.** Immunohistological pattern between different generations. **Table S5.** mRNA profile pathway analysis between P0 and P2 (FC>2.0). **Figure S1.** High density tissue microarrays (TMA) and immunohistochemistry conducted in primary and xenograft high-grade serous carcinoma. (DOCX 376 kb)

## Abbreviations

PDX: Patient derived xenografts
HGSC: High-grade serous carcinoma
EOC: Epithelial ovarian cancer
Taxol/Carbo: Paclitaxel and carboplatin
RIN: RNA integrity number
qPCR: Quantitative real-time PCR
NOD: Nonobese diabetic
SC: Subcutaneous
IB: Intrabursa
DAB: Diaminobenzidine
FFPE: Formalin-fixation and paraffin-embedding
TMA: Tissue microarray
SOE: Success of engraftment
N/C ratio: Nuclear cytoplasmic ratio
GVHD: Graft-versus-host disease
CAMs: Cell adhesion molecules
SR: Subrenal
IP: Intraperitoneal
CSC: Cancer stem cell
