# Supplementary material for: Histologic and molecular analysis of patient derived xenografts of high-grade serous ovarian carcinoma
Source: J Hematol Oncol. 2016 Sep 21;9:92. doi: 10.1186/s13045-016-0318-6 (PMC5031262; doi:10.1186/s13045-016-0318-6)

**Additional file 1**

**Table S1 Primers and sequences used in this study**

Primer name Forward Sequence (5’-3’) Reverse Sequence (5’-3’)

U6 AACGCTTCACGAATTTGCGT Universal

P53-exon4 AATGGATGATTTGATGCTGTCCC CTCAGGGCAACTGACCGTGC

P53-exon5 TTCAACTCTGTCTCCTTCCT CAGCCCTGTCGTCTCTCCAG

P53-exon6 GCCTCTGATTCCTCACTGAT TTAACCCCTCCTCCCAGAGA

P53-exon7 AAGGCGCACTGGCCTCATCTT GCACAGCAGGCCAGTGTGCAG

P53-exon8 TTCCTTACTGCCTCTTGCTT CGCTTCTTGTCCTGCTTGCT

P53-exon9 CAAGGGTGCAGTTATGCCT ACTTGATAAGAGGTCCCAA

**Table S2 Information of antibodies used in IHC assay**

Antibody Vendor Catalog number Concentration

ALDH1 BD 611195 1;1000

CD24 NeoMarkers MS-1278-P1 1;200

CD133 Miltenyl Biotec 130-090-422 1;20

ER Thermo RM-9101S 1;200

HMGA2 BioCheck 59170AP 1;500

KI-67 Dako M7240 1;100

MTSS1 Novus H00009788-M01 1;100

P16 Santa Cruz SC:81157 1;200

P21 Dako M7202 1;50

P53 Dako M7001 1;200

PR Dako M3569 1;1600

**Table S3 Histology pattern between different passages**

| Case ID | Generation | Nucleus grade | Mitosis (/10HPF) | Necrosis (%) | N/C ratio | Stroma (%) |
| --- | --- | --- | --- | --- | --- | --- |
| OVCA4 | Total | 3±0 | 21.47±1.66 | 14.91±1.86 | 2.28±0.08 | 12.42±1.18 |
|  | P0 | 3±0 | 12.50±4.79 | 10±0 | 2.75±0.25 | 25±9.57 |
|  | P1 | 3±0 | 31.83±3.37 | 33.33±4.22 | 2.67±0.21 | 15±2.24 |
|  | P2 | 3±0 | 22±2.34 | 11.56±1.87 | 2.13±0.15 | 10.63±1.01 |
|  | P3 | 3±0 | 18.89±4.39 | 13.44±5.41 | 2±0 | 10.44±0.84 |
|  | P4 | 3±0 | 20±3.27 | 11.88±2.49 | 2.38±0.18 | 10±1.64 |
| OVCA5 | Total | 2.93±0.07 | 25±3.31 | 5.67±2.88 | 2.73±0.12 | 18.33±2.83 |
|  | P0 | 2.50±0.50 | 32.50±7.50 | 20±20 | 2.50±0.50 | 20±0 |
|  | P1 | 3±0 | 30±0 | 5±5 | 3±0 | 7.50±2.50 |
|  | P2 | 3±0 | 22.73±4.23 | 3.18±1.94 | 2.73±0.14 | 20±3.57 |
| OVCA6 | Total | 3±0 | 33.75±8 | 10±3.54 | 2.75±0.25 | 10±3.54 |
|  | P0 | 3 | 45 | 20 | 3 | 10 |
|  | P1 | 3±0 | 35±15 | 7.50±2.50 | 3±0 | 5±0 |
|  | P2 | 3 | 20 | 5 | 2 | 20 |
| OVCA7 | Total | 2.75±0.14 | 25.50±8.18 | 10±8.66 | 3±0 | 15±6.12 |
|  | P0 | 2.75±0.25 | 35±5 | 15±15 | 3±0 | 5±0 |
|  | P1 | 2.75±0.25 | 16±14 | 0 | 3±0 | 25±5 |
| OVCA8 | Total | 2.77±0.08 | 20.10±2.42 | 16.48±4.71 | 2.27±0.13 | 12.33±1.60 |
|  | P0 | 3±0 | 8.50±6.50 | 10±10 | 2±0 | 25±5 |
|  | P1 | 3±0 | 30±7.07 | 12.50±4.79 | 2.25±0.25 | 16.25±2.39 |
|  | P2 | 3±0 | 17.50±3.13 | 20.63±6.30 | 2±0.27 | 10±0 |
|  | P3 | 2.63±0.13 | 15±4.33 | 26.25±23.04 | 2.25±0.25 | 14.67±6.64 |
|  | P4 | 2.13±0.13 | 25±5 | 5.63±2.13 | 3±0 | 5±0 |
| OVCA9 | Total | 2.75±0.14 | 32±10.56 | 3.75±2.39 | 3±0 | 23.13±4.25 |
|  | P0 | 3±0 | 47.50±2.50 | 2.50±2.50 | 3±0 | 25±5 |
|  | P1 | 2.50±0 | 16.50±13.50 | 5±5 | 3±0 | 21.25±8.75 |
| OVCA10 | Total | 2.67±0.33 | 1.83±0.60 | 36.67±23.33 | 1.83±0.17 | 26.67±12.02 |
|  | P0 | 2 | 3 | 0 | 2 | 50 |
|  | P1 | 3±0 | 1.25±0.25 | 55±25 | 1.75±0.25 | 15±5 |
| OVCA13 | Total | 3±0 | 51.67±12.76 | 18.33±7.92 | 3±0 | 31.67±11.30 |
|  | P0 | 3±0 | 57.50±19.31 | 7.50±4.79 | 3±0 | 45±11.90 |
|  | P1 | 3±0 | 40±0 | 40±10 | 3±0 | 5±0 |

**Table S4 Immunohistological pattern between different generations**

| Case ID | Generation | P53  （%） | P16 （Intensity） | ER（%） | PR（%） | Ki67 (%) | P21  （%） | ALDH1 (%) | CD24 (%) | CD133 (%) | HMGA2 (Intensity) |
| --- | --- | --- | --- | --- | --- | --- | --- | --- | --- | --- | --- |
| OVCA4 | Total | 90±2.18 | 1.34±0.09 | 20.58±3.38 | 3.81±1.06 | 45.81±3 | 17.44±1.79 | 4.95±1.07 | 46.53±3.23 | 11.65±318 | 1.58±0.14 |
|  | P0 | 95±0 | 1.75±0.25 | 47.50±6.29 | 10.00±7.07 | 47.50±16.52 | 20±8.90 | 22.5±2.5 | 50±7.07 | 16.50±8.84 | 2±0.41 |
|  | P1 | 80±10.25 | 1.83±0.17 | 11.67±4.59 | 2.67±1.67 | 48.33±6.54 | 15±3.42 | 3±0.89 | 38.33±7.92 | 4.42±1.40 | 2±0.63 |
|  | P2 | 87.19±4.18 | 1.28±0.14 | 11.56±3.19 | 4.88±1.93 | 54.38±4.08 | 19.38±2.95 | 2.19±0.73 | 41.94±6.44 | 8.84±4.20 | 1.44±0.22 |
|  | P3 | 95±0 | 1.11±0.11 | 14.44±7.04 | 2.78±1.47 | 26.67±4.08 | 15.56±4.60 | 6.89±1.95 | 51.11±7.35 | 12.56±8.49 | 1.44±0.29 |
|  | P4 | 95±0 | 1.13±0.23 | 38.75±10.25 | 0.63±0.63 | 47.50±5.90 | 16.25±3.63 | 1±0.60 | 55±4.23 | 19.25±11.09 | 1.5±0.19 |
| OVCA5 | Total | 91.67±3.71 | 1.67±0.17 | 65.38±5.01 | 2.17±0.85 | 30.83±3.92 | 8.83±1.54 | 5.42±1.56 | 16.67±3.91 | 13.42±5.30 | 1.25±0.22 |
|  | P0 | 95±0 | 1.50±0.50 | 70±10 | 0±0 | 27.50±22.50 | 3±2 | 5.50±4.50 | 5±0 | 20.50±19.50 | 2±1 |
|  | P1 | 97.50±2.50 | 1.50±0.50 | 75±5 | 2.5±2.5 | 22.50±7.50 | 12.50±7.50 | 3±2 | 5±0 | 7.50±2.50 | 1±1 |
|  | P2 | 89.38±5.25 | 1.75±0.21 | 62.22±6.70 | 2.63±1.12 | 33.75±3.93 | 9.38±1.49 | 6±2.10 | 22.50±4.70 | 13.13±6.93 | 1.13±0.19 |
| OVCA6 | Total | 97.5±1.44 | 3±0 | 53.75±17.72 | 2.75±1.31 | 67.50±8.54 | 21.25±8.26 | 41.25±11.97 | 36.25±12.14 | 34±19.58 | 1±0.41 |
|  | P0 | 95±0 | 3±0 | 50±0 | 0±0 | 60±0 | 30±0 | 70±0 | 30±0 | 1±0 | 2±0 |
|  | P1 | 100±0 | 3±0 | 80±0 | 3±2 | 80±10 | 25±15 | 22.50±7.50 | 27.50±22.50 | 22.50±7.50 | 0.5±0.5 |
|  | P2 | 95±0 | 3±0 | 5±0 | 5±0 | 50±0 | 5±0 | 50±0 | 60±0 | 90±0 | 1±0 |
| OVCA7 | Total | 3.33±2.89 | 2.5±0.29 | 86.25±3.75 | 0±0 | 35±13.23 | 7.50±1.44 | 5.5±2.60 | 67.50±6.29 | 51.25±20.25 | 0.50±0.50 |
|  | P0 | 5±5 | 2.5±0.5 | 87.50±7.50 | 0±0 | 30±10 | 7.50±2.50 | 10±0 | 75±5 | 60±30 | 1±1 |
|  | P1 | 0±0 | 2.5±0.5 | 85±5 | 0±0 | 40±30 | 7.50±2.50 | 1±0 | 60±10 | 42.50±37.50 | 0±0 |
| OVCA8 | Total | 2.86±1.45 | 2.50±0.11 | 1.91±1.41 | 0.09±0.06 | 31.43±4.62 | 14.09±1.85 | 1.50±0.66 | 25.95±2.89 | 29.29±3.91 | 1.86±0.22 |
|  | P0 | 15±15 | 2.50±0.50 | 5±5 | 0±0 | 30±20 | 15±5 | 2.50±2.50 | 40±10 | 45±25 | 3±0 |
|  | P1 | 2.50±1.44 | 2.50±0.29 | 0±0 | 0±0 | 30±4.08 | 20±0 | 3±2.35 | 27.50±4.79 | 27.50±2.50 | 1.75±0.48 |
|  | P2 | 1.43±0.86 | 2.50±0.19 | 0.25±0.16 | 0.25±0.16 | 31.88±9.82 | 15.63±4.27 | 0.75±0.62 | 14.38±3.95 | 18.13±3.77 | 1±0.33 |
|  | P3 | 0±0 | 2.50±0.29 | 0±0 | 0±0 | 11.67±3.82 | 12.50±2.50 | 2.50±2.50 | 36.67±2.89 | 46.67±15.28 | 2.75±0.25 |
|  | P4 | 2.50±2.50 | 2.50±0.29 | 7.5±7.5 | 0±0 | 47.50±7.50 | 6.25±1.25 | 0±0 | 32.50±4.79 | 32.50±4.79 | 2.25±0.25 |
| OVCA9 | Total | 2.50±2.50 | 3±0 | 27.50±11.09 | 3.75±2.39 | 57.50±13.15 | 7.50±1.44 | 7.75±4.19 | 60±5.77 | 51.25±18.75 | 1.50±0.65 |
|  | P0 | 5±5 | 3±0 | 30±10 | 7.5±2.5 | 80±0 | 7.50±2.50 | 12.50±7.50 | 60±10 | 57.50±37.50 | 2.50±0.50 |
|  | P1 | 0±0 | 3±0 | 25±25 | 0±0 | 35±5 | 7.50±2.50 | 3±2 | 60±10 | 45±25 | 0.50±0.50 |
| OVCA10 | Total | 0±0 | 2.67±0.33 | 23.33±23.33 | 0±0 | 10±0 | 3.33±1.67 | 1.67±1.67 | 26.67±3.33 | 6.67±1.67 | 2±0.58 |
|  | P0 | 0±0 | 2±0 | 0±0 | 0±0 | 10±0 | 5±0 | 5±0 | 30±0 | 5±0 | 3±0 |
|  | P1 | 0±0 | 3±0 | 35±35 | 0±0 | 10±0 | 2.50±2.50 | 0±0 | 25±5 | 7.50±2.50 | 1.50±0.50 |
| OVCA13 | Total | 1.67±1.67 | 3±0 | 30.83±9.52 | 3.50±2.06 | 32.50±9.64 | 10±2.23 | 25±6.19 | 60±10.95 | 38.75±14.75 | 1.83±0.40 |
|  | P0 | 2.50±2.50 | 3±0 | 33.75±10.68 | 5.25±2.75 | 31.25±15.05 | 11.25±3.15 | 25±9.57 | 70±11.55 | 70±0 | 2±0.58 |
|  | P1 | 0±0 | 3±0 | 25±25 | 0±0 | 35±5 | 7.50±2.50 | 25±5 | 40±20 | 7.50±2.50 | 1.50±0.50 |

**Table S5 mRNA profile pathway analysis between P0 and P2 (FC>2.0)**

| Pathway | P Value | Genes | Fold enrichment |
| --- | --- | --- | --- |
| hsa05310:Asthma | 3.98E-10 | LOC100133678, HLA-DRB3, HLA-DRB4, FCER1G, LOC100133583, HLA-DPA1, HLA-DOA, HLA-DMA, HLA-DQA1, HLA-DRA | -28.18041872 |
| hsa05330:Allograft rejection | 7.66E-08 | LOC100133678, HLA-DRB3, HLA-DRB4, LOC100133583, HLA-DPA1, HLA-DOA, HLA-DMA, HLA-DQA1, HLA-DRA | -20.17857143 |
| hsa05332:Graft-versus-host disease | 1.38E-07 | LOC100133678, HLA-DRB3, HLA-DRB4, LOC100133583, HLA-DPA1, HLA-DOA, HLA-DMA, HLA-DQA1, HLA-DRA | -18.62637363 |
| hsa04940:Type I diabetes mellitus | 2.36E-07 | LOC100133678, HLA-DRB3, HLA-DRB4, LOC100133583, HLA-DPA1, HLA-DOA, HLA-DMA, HLA-DQA1, HLA-DRA | -17.29591837 |
| hsa04672:Intestinal immune network for IgA production | 7.08E-07 | LOC100133678, HLA-DRB3, HLA-DRB4, LOC100133583, HLA-DPA1, HLA-DOA, HLA-DMA, HLA-DQA1, HLA-DRA | -14.82507289 |
| hsa05320:Autoimmune thyroid disease | 9.38E-07 | LOC100133678, HLA-DRB3, HLA-DRB4, LOC100133583, HLA-DPA1, HLA-DOA, HLA-DMA, HLA-DQA1, HLA-DRA | -14.24369748 |
| hsa05416:Viral myocarditis | 4.58E-08 | LOC100133678, RAC2, HLA-DRB3, HLA-DRB4, LOC100133583, ITGB2, HLA-DPA1, HLA-DOA, HLA-DMA, HLA-DQA1, HLA-DRA | -12.78923541 |
| hsa05020:Prion diseases | 0.006135942 | C1QB, PRNP, CCL5, C1QC | -10.37755102 |
| hsa04512:ECM-receptor interaction | 2.56E-06 | COL6A3, COL3A1, COL1A2, COL6A1, COL1A1, COL5A2, THBS2, COL5A1, SPP1 | -9.728954082 |
| hsa05322:Systemic lupus erythematosus | 8.44E-07 | LOC100133678, C1QB, HLA-DRB3, HLA-DRB4, LOC100133583, HLA-DPA1, HLA-DOA, C1QC, HLA-DMA, HLA-DQA1, HLA-DR | -9.172077922 |
| hsa04514:Cell adhesion molecules (CAMs) | 9.61E-09 | HLA-DRB3, ITGB2, LOC100133583, CLDN11, HLA-DMA, HLA-DQA1, LOC100133678, PECAM1, CD2, HLA-DRB4, HLA-DPA1, HLA-DOA, JAM3, HLA-DRA | -8.942775974 |
| hsa04612:Antigen processing and presentation | 2.58E-05 | LOC100133678, HLA-DRB3, HLA-DRB4, LOC100133583, HLA-DPA1, HLA-DOA, HLA-DMA, HLA-DQA1, HLA-DRA | -8.752151463 |
| hsa05130:Pathogenic Escherichia coli infection | 0.023219364 | LY96, WAS, TUBB3, CD14 | -6.372180451 |
| hsa04640:Hematopoietic cell lineage | 0.002203706 | HLA-DRB3, CD2, HLA-DRB4, CD14, HLA-DRA, CSF1R | -6.33513289 |
| hsa05214:Glioma | 0.030120403 | CDKN1A, PDGFRA, PDGFRB, CDK6 | -5.765306122 |
| hsa04510:Focal adhesion | 7.27E-06 | RAC2, COL6A3, COL3A1, COL1A2, PDGFRA, PDGFRB, COL6A1, COL1A1, COL5A2, THBS2, COL5A1, SPP1 | -5.421108742 |
| hsa04670:Leukocyte transendothelial migration | 0.001575607 | RAC2, MMP9, PECAM1, ITGB2, CLDN11, JAM3, THY1 | -5.386652542 |
| hsa05218:Melanoma | 0.040824219 | CDKN1A, PDGFRA, PDGFRB, CDK6 | -5.115694165 |
| hsa04620:Toll-like receptor signaling pathway | 0.095216939 | LY96, CCL5, CD14, SPP1 | -3.596181047 |
| hsa04810:Regulation of actin cytoskeleton | 0.027450803 | RAC2, ARHGEF6, PDGFRA, PDGFRB, ITGB2, WAS, CD14 | -2.956395349 |

**Figure S1** High density tissue microarrays (TMA) and immunohistochemistry conducted in primary and xenograft high-grade serous carcinoma.


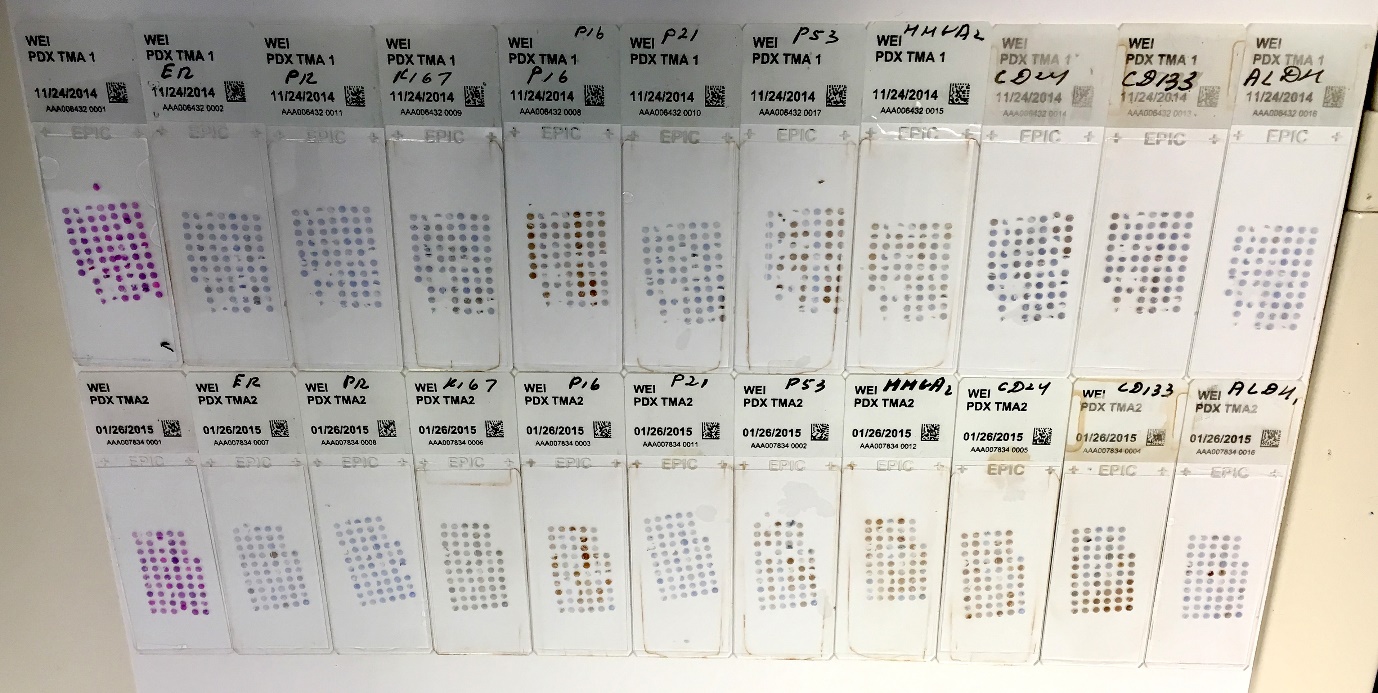

Supplement: Additional file 1: Table S1. — Primers and sequences used in this study. Table S2. Information of antibodies used in IHC assay. Table S3. Histology pattern between different passages. Table S4. Immunohistological pattern between different generations. Table S5. mRNA profile pathway analysis between P0 and P2 (FC>2.0). Figure S1. High density tissue microarrays (TMA) and immunohistochemistry conducted in primary and xenograft high-grade serous carcinoma. (DOCX 376 kb) [file 13045_2016_318_MOESM1_ESM.docx]
